# Left–right synchronicity of vagal spikes and their coordination with peripheral and cortical activity in rats

**DOI:** 10.3389/fncir.2026.1944995

**Published:** 2026-10-30

**Authors:** Reina Shiratori, Nahoko Kuga, Takuya Sasaki

**Affiliations:** 1 Department of Pharmacology, Graduate School of Pharmaceutical Sciences, Tohoku University, Sendai, Japan; 2 Division of Creative Interdisciplinary Research, Frontier Research Institute for Interdisciplinary Sciences, Tohoku University, Sendai, Japan; 3 Department of Neural Healthspan, Healthspan Research Center (HeSReC), Tohoku University, Sendai, Japan; 4 Department of Neuropharmacology, Tohoku University School of Medicine, Sendai, Japan

**Keywords:** gastrointestinal motility, heart rate, prefrontal cortex, spike recording, vagus nerve

## Abstract

The vagus nerve (VN) serves as a major communication pathway between the brain and peripheral organs. The left and right VN exhibit asymmetric structures and are considered to sense diverse peripheral organ states. However, it remains unknown how coordinately the left and right VN generate physiological spike activity. Here, we performed simultaneous electrophysiological recordings of left and right cervical VN spike patterns in rats. During non-movement periods, ongoing spike activity in both VNs was predominantly organized into burst events. The occurrence timing of burst events was partially synchronized between the left and right VN, whereas individual spike timing within burst events showed no pronounced synchronization. Under anesthetized conditions, VN spike activity was not strongly entrained to oscillatory gastric or intestinal motility on a cycle-by-cycle basis but pharmacological enhancement of gastrointestinal activity was sufficient to induce bilaterally synchronized burst events in a pattern closely resembling that observed under freely moving conditions. During VN burst events in non-movement periods, prefrontal cortical local field potential power across the wide frequency range was substantially modulated, suggesting a functional link between vagal afferent activity and higher-order brain dynamics. Taken together, these findings advance our insight into VN-mediated brain–body interactions.

## Introduction

The afferent vagus nerve (VN) transmits ascending interoceptive signals from visceral organs, including the heart and gastrointestinal tract, to the brain. Accumulating evidence demonstrates that the vagal interoceptive signals have been shown to sustain emotional states (Bravo et al., 2011; Liu and Forsythe, 2021; Okonogi et al., 2024) and vagus nerve stimulation (VNS) improves mental states for psychiatric symptoms (Rush et al., 2000; Nemeroff et al., 2006; George et al., 2008; Furmaga et al., 2011; Austelle et al., 2024) and diverse cognitive functions such as learning, memory, and decision-making (Pena et al., 2013; Noble et al., 2017; Suarez et al., 2018; Neuser et al., 2020; Bowles et al., 2022; Olsen et al., 2023; Martin et al., 2024; Teckentrup and Kroemer, 2024).

The VN runs bilaterally along the left and right sides of the body, and the left and right VN innervate peripheral organs in patterns that are partially distinct and overlapping (Neuhuber and Berthoud, 2021; Bassi et al., 2022). In the heart, the right VN projects predominantly to the sinoatrial (SA) node, and its stimulation induces pronounced bradycardia, whereas the left VN preferentially innervates the atrioventricular (AV) node and has a weaker influence on the SA node, an organization described across species from rodents to humans (Ardell and Randall, 1986; Schiereck et al., 2000; Kronsteiner et al., 2024). Furthermore, vagal firing responds asymmetrically to unilateral injury in minipigs, as left cardiac vagotomy reduces spike rates in both the transected left vagus and the intact right vagus, whereas right cardiac vagotomy does not strongly affect contralateral spikes (Vallone et al., 2024). In the gastrointestinal tract, owing to its left–right asymmetrical structure, VN innervation exhibits clear asymmetrical patterns. The left afferent VN projects more densely to the ventral stomach and the ventral proximal duodenum, whereas the right VN projects to the dorsal side of these regions and more densely innervates the jejunum/ileum and colon (Berthoud et al., 1997; Wang and Powley, 2000; Wang and Tache, 2024; Lansbury et al., 2025). Furthermore, the molecular expression pattern of the VN is highly heterogeneous (Zhao et al., 2022; Lansbury et al., 2025). For example, the left VN shows higher expression of TRPV1 receptors (responsive to capsaicin), whereas the right VN shows higher expression of CCKR1 receptors (responsive to CCK), and baseline calcium activity in the left nodose ganglion (NG) is higher than in the right NG (Ritchey et al., 2026).

The vagal asymmetry at the anatomical and molecular levels implies that the left and right VN may be differentially activated in response to peripheral organ states, conveying distinct interoceptive signals to the brain (Bassi et al., 2022). Indeed, left and right VNS have been reported to exert distinct effects on brain functions. Left VNS has been shown to mainly modulate cognitive functions, including emotion and memory (Nemeroff et al., 2006; Noble et al., 2017; Noble et al., 2019; Okonogi et al., 2024), and feeding behavior (de Araujo et al., 2023), whereas the right VNS has been proposed to more readily activate brain reward circuits (Han et al., 2018; Brougher et al., 2021; de Araujo et al., 2023). These lines of evidence highlight left–right differences as a critical factor in understanding the functional significance of vagal activity.

However, it remains to be resolved how the left and right VNs cooperatively or independently generate their nerve activity and thereby influence brain functions. To address these physiological issues, we established simultaneous spike recordings from the bilateral VN in freely moving rats and analyzed whether the left and right VN exhibit similar or distinct ongoing spike patterns, whether they engage in coordinated synchronous activity, and how such activity influences downstream brain activity rhythms (e.g., local field potential (LFP)). In addition, to examine the detailed relationship between VN spike patterns and peripheral organ activity, we simultaneously recorded gastrointestinal activity, including electrogastrogram (EGG) and electroenterogram (EEnG) signals, and tested the effects of pharmacological organ activation under anesthetized conditions.

## Materials and methods

### Approvals

All experiments were performed with the approval of the Committee on Animal Experiments at Tohoku University (approval number: 2022 PhA-004) and in accordance with the NIH guidelines for the care and use of animals. All experiments were reported in compliance with the ARRIVE guidelines.

### Subjects

Male Wistar rats (2 to 4 months old) with weights of 230–330 g were used in this study. All rats were purchased from SLC (Shizuoka, Japan) and maintained with free access to water and food under a 12-h light/12-h dark schedule under housing conditions at 23 °C ± 1 °C and relative humidity of 50 ± 5% with lights off at 8:00 p.m. Female rats were excluded because gastrointestinal motility varies across the estrous cycle (Jiang et al., 2019).

### Surgery for electrophysiological recording in freely moving conditions

Rats were anesthetized with 1.0%–2.5% isoflurane gas while the rats lay on their backs. Veterinary ointment was applied to the rats’ eyes to prevent dryness. For all steps of an incision, the skin at the incision site was sterilized with betadine and 70% ethanol. The following surgeries for electrode implantation were performed as described previously (Shikano et al., 2018; Kuga et al., 2019; Shikano et al., 2019).

For implanting VN electrodes, an incision was made in the left/right neck area from the larynx to the sternum, and the bundle including the VN and the carotid artery was isolated. A custom-made flat-plate bipolar silver electrode (1.0 × 3.0 mm per pole; interelectrode gap = 0.3 mm) was placed underneath the isolated cervical VN, and the VN together with the electrode was secured with a silicone elastomer (Kwik-Cast, World Precision Instruments, Sarasota, FL, USA). A reference electrode (stainless-steel wires; AS 633, Cooner Wire Company), electrically insulated except at the tip, was placed on the salivary gland immediately above the VN electrode to minimize noise artifacts at the recording site. The free ends of the electrodes were externalized through the incision.

For implanting electromyogram (EMG) electrodes, the rat was fixed in a stereotaxic instrument with two ear bars and a nose clamp, and a midline incision was made in the skin from the skull to the dorsal neck area. Two EMG electrodes (AS633, Cooner Wire Company, CA, USA) were sutured to the dorsal neck area.

For implanting electrocardiogram (ECG) electrodes, an incision was made on the medial chest area, and the intercostal muscles were exposed by separating the chest muscles. A ring-type stainless-steel ECG electrode (AS633, Cooner Wire Company, CA, USA) was attached to the intercostal muscles on the left side of the chest.

For implanting LFP electrodes, a midline incision was made above the skull and circular craniotomies with a diameter of 1.0 mm were created using a high-speed drill at coordinates of both sides of the PFC (3.2 mm anterior, 0.8 mm bilateral and 1.2 mm depth to bregma) and stainless-steel screw-shaped electrodes with a diameter of 1.0 mm were stereotaxically implanted above the prefrontal cortex (PFC) (Abe et al., 2019). In addition, the same stainless-steel screw-shaped electrodes were implanted on the cerebellum as an earth/reference.

The free ends of the VN, EMG, ECG, LFP, and earth/reference electrodes were soldered to the protruding insulated wires corresponding to each channel on the electrode interface board (EIB). All the wires and the electrode assembly were secured to the skull using dental cement. After all surgical procedures were completed, anesthesia was discontinued, and the rats were allowed to awaken spontaneously. Following surgery, each animal was housed individually in a transparent Plexiglas home cage (25 cm × 40 cm) with free access to water and food. All the recordings were conducted in the home cage after at least 1 week following the surgery.

### Surgery for acute electrophysiological recording under anesthetized conditions

Rats were anesthetized with urethane (1.0 g/kg, i.p.) and placed in the supine position. Veterinary ointment was applied to the rats’ eyes to prevent dryness. For all steps of an incision, the skin at the incision site was sterilized with betadine and 70% ethanol.

VN, EMG, and ECG electrodes were implanted using the same procedure as that used for the freely moving recording surgery.

For implanting EGG and EEnG electrodes, a left abdominal incision was made, and a stainless-steel electrode (AS633, Cooner Wire Company, CA, USA) was sutured to the gastric and jejunal serosa, respectively (Shiratori et al., 2024).

For LFP electrode implantation, stainless-steel screw electrodes were stereotaxically implanted above the PFC and the cerebellum (as a reference) using the same procedure as described above.

The free ends of all electrodes were soldered to the corresponding channels on the EIB, and all the wires and the electrode assembly were secured to the skull using dental cement.

To apply drugs to the organs, 0.3 mL of acetylcholine (1.0 mg/mL) or isoproterenol (0.5 mg/mL) dissolved in phosphate-buffered saline (PBS; pH 7.4) was topically applied to the gastrointestinal tract (stomach or intestine) and heart, respectively. To block the effects of each agonist, 0.3 mL of atropine (0.1 mg/mL) or propranolol (2.0 mg/mL) was applied to the same site 15 min after drug application.

For vagotomy, the bilateral cervical VNs were exposed and dissected by surgical micro scissors.

### Electrophysiological recording

The rat was connected to the recording system via the Cereplex M headstage (Blackrock Neurotech, UT, USA), a digitally programmable amplifier attached near the head. The output of the headstage was transmitted to the Cereplex Direct recording system, a data acquisition system, through a lightweight multiwire tether and a commutator. VN signals were sampled at 30 kHz and high-pass filtered at 100 Hz. LFP, EEnG, EGG, and EMG signals were sampled at 2 kHz and low-pass filtered at 500 Hz.

### Histological analysis

The rat was overdosed with isoflurane, transcardially perfused with PBS followed by 4% paraformaldehyde in PBS, and decapitated. After dissection, the brains were post-fixed overnight in 4% paraformaldehyde and equilibrated with 30% sucrose in PBS overnight at 4 °C. Frozen coronal sections (50 μm thick) were cut using a microtome (LEICA SM2010 R; Leica Microsystems, Wetzlar, Germany), and serial sections were mounted and processed for cresyl violet staining. For cresyl violet staining, the slices were rinsed in water, stained with cresyl violet, and coverslipped with hydrophobic mounting medium (Marinol). The positions of the LFP electrodes were confirmed using an optical microscope (All-in-One Fluorescence Microscope BZ-X710, Keyence Corporation, Osaka, Japan).

### Data analysis

To reduce background electrical noise and increase the signal-to-noise ratio (Sahin and Durand, 1998; Caravaca et al., 2017; Silverman et al., 2018; Okonogi et al., 2024), VN signals were subtracted from signals from a reference electrode placed on the salivary gland and high-pass filtered at 100 Hz. Non-movement periods were extracted by manually setting a threshold on EMG root-mean-square (RMS) traces. During these periods a spike unit was detected when the amplitude of a negative deflection of the filtered VN signals exceeded a threshold that was manually adjusted for individual rats. Basically, the thresholds were set at approximately three standard deviations (SDs) below the mean of the baseline-filtered VN signal. The exact threshold for each recording was determined by the experimenter based on visual inspection of individual spike-like waveforms relative to the noise level. In ECG traces, R-wave peaks were identified. In some rats, VN recordings contained ECG-like signals occurring at a constant interval of 130–170 ms; these components were excluded by applying an amplitude threshold, and signals below this threshold were not detected. In VN spike trains, sparse single spikes were defined as spikes preceded and followed by inter-spike intervals (ISIs) of >3 s. Burst events were defined as >30 consecutive spikes with ISIs shorter than 100 ms. VN spike rates in each time bin were z-scored based on the mean and SD of spike rates across the entire recording period.

For EGG/EEnG analysis, signals were analyzed using fast Fourier transform (FFT) in the 0–1 Hz frequency band.

To compute the time-frequency representation of LFP power in the 2–100 Hz frequency band, LFP signals were convolved using complex Morlet wavelet transformation by MATLAB R2024b. The signals that included apparent electrical noise due to physical striking of the animal’s head to the walls were manually removed. Behavioral states were classified from the LFP spectrum averaged within non-overlapping 4-s epochs (Watson et al., 2016). For each epoch, three features were calculated. A slow-wave index was defined as the logarithm of the ratio of delta (0.5–4 Hz) to gamma (30–80 Hz) power. A theta ratio was defined as the logarithm of the ratio of theta (6–10 Hz) to delta power. The logarithm of high-frequency power (>100 Hz) was used as a proxy for muscle activity. Each feature was smoothed with a Gaussian kernel spanning three epochs. Classification thresholds were determined separately for each rat by fitting a two-component Gaussian mixture model to the distribution of each feature and taking the value at which the posterior probabilities of the two components were equal. Epochs with a slow-wave index above threshold were classified as NREM sleep. Among the remaining epochs, those with high-frequency power above threshold were classified as active wakefulness. Of the remaining low-activity epochs, those with a theta ratio above threshold were classified as REM sleep, and the others were classified as quiet wakefulness. Bouts shorter than three consecutive epochs (12 s) were reassigned to the preceding state, and REM episodes not preceded by NREM sleep were reclassified as quiet wakefulness.

### Statistical analysis

All data were presented as the mean ± standard error of the mean (SEM), unless otherwise specified, and were analyzed using MATLAB R2024b. For comparisons, within-subject data were analyzed by paired *t*-test. Multiple group comparisons were corrected using *post hoc* FDR correction. Correlations between left and right VN spike rates were assessed using Pearson’s correlation coefficient. Synchrony of burst events was evaluated by comparing the observed values with a null distribution generated from 1,000 shuffled surrogate datasets. For drug-response analyses, spike-rate Z-scores were calculated relative to the pre-drug baseline, and responses with Z-scores exceeding the threshold corresponding to *p* < 0.01 were considered significant. Statistical significance was defined as *p* < 0.05.

## Results

### Simultaneous recording of left and right VN spikes

Rats were implanted with bipolar electrodes on the left and right VN, an EMG electrode on the dorsal neck muscle, and an ECG electrode on the intercostal muscle (Figures 1A,B). After recovery from surgery for a week, electrophysiological recordings were performed under freely moving conditions in a homecage. The recording duration was 8,994 ± 1,073 s (range, 5,312–13,526 s; median, 7,720 s; *n* = 9 rats). Representative traces recorded from a rat are shown in Figure 1C. The magnitude of EMG signals was quantified by computing their RMS (root mean square) (bin = 1 s). Periods in which the EMG RMS exceeded a threshold (~5 × SD above the average baseline) were defined as move periods. These periods were excluded from VN spike analysis, as they contained large spike-like artifacts or movement-derived noise (Figure 1C, move). During non-movement periods with smaller EMG amplitudes [6,064 ± 1,069 s (range, 1985–12,643 s; median, 5,914 s; *n* = 9 rats)], VN signals were composed of complex compound extracellular signals from VN fibers (Steinberg et al., 2016; Silverman et al., 2018; Okonogi et al., 2024; Miki et al., 2025). The timing of spike units was detected when the amplitude of the negative deflection in the filtered VN signal exceeded a manually defined threshold (~3 × SD below the average) during the other non-movement periods (Figure 1D; magnified traces showing that VN spikes occur independently of EMG traces). The distribution of peak-to-peak amplitudes, defined as the difference between the positive and negative peaks, showed that most spikes had small amplitudes, whereas large-amplitude spikes occurred with low probability (Figure 1E). These detected spikes represent the summation of action potentials arising from multiple axonal fibers and can therefore be regarded as multiunit activity. Based on anatomical observations that 75–90% of the fibers in the cervical VN are afferent fibers (Evans and Murray, 1954; Agostoni et al., 1957; Prechtl and Powley, 1990; Miki et al., 2025), the majority of VN signals are considered to represent afferent fiber activity. In some rats, VN signals contained sharp positive deflections that were closely locked to the heartbeat with a constant interval of 130–170 ms (Figure 1F). For detecting VN spikes, these heartbeat-locked components were excluded by setting an amplitude threshold below which signals were not detected. Overall, the mean spike counts of the left and right VN during non-movement periods were 1.3 ± 1.3 and 1.9 ± 2.4 counts/s, respectively (range, 0.3–4.6 and 0.04–7.2 counts/s; median, 0.79 and 0.59 counts/s; *n* = 9 rats).

**Figure 1:**
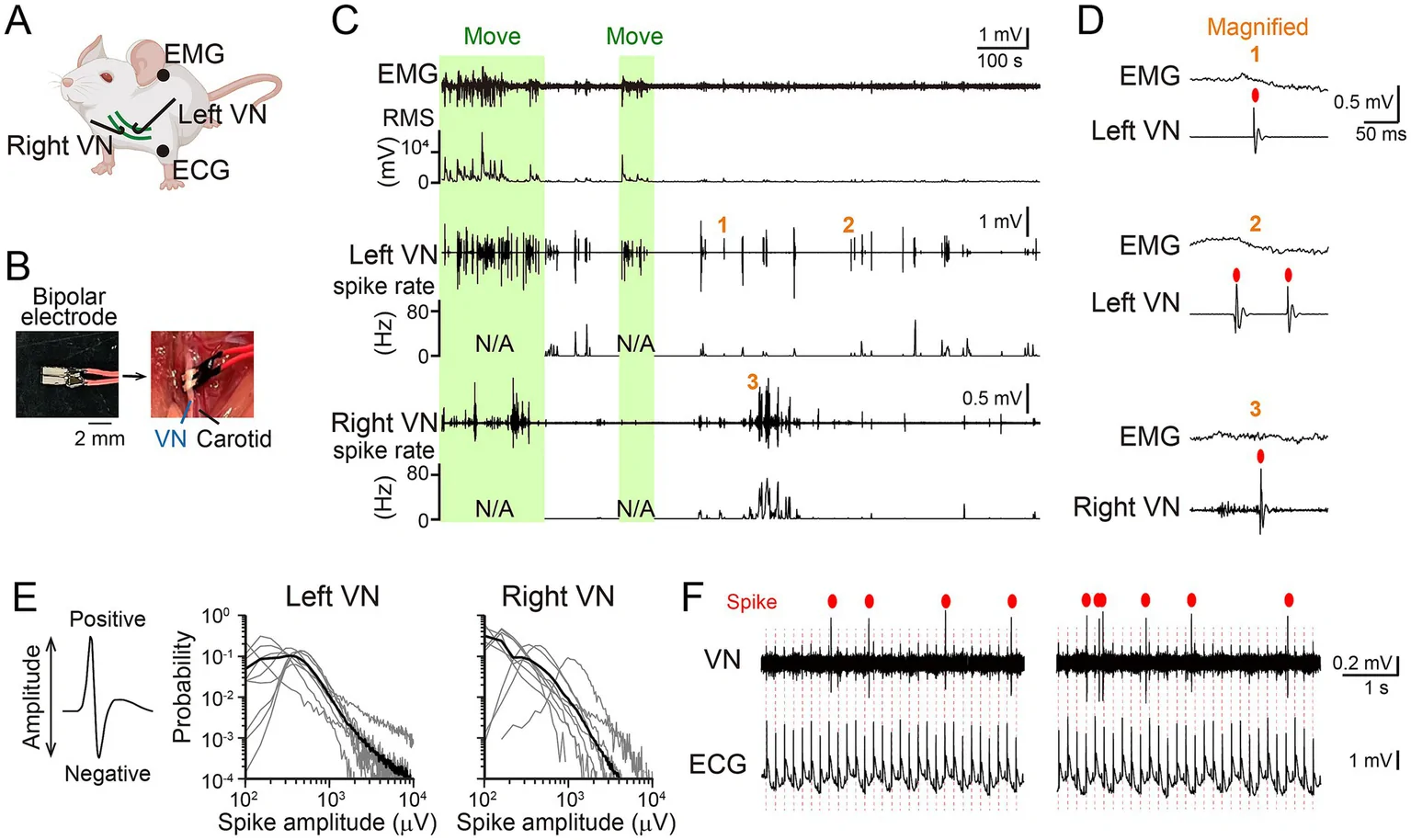
Recording of left–right vagal spikes in rats. **(A)** Simultaneous recordings from the left and right VN using bipolar electrodes in a freely moving rat. ECG and EMG signals were also simultaneously recorded. **(B)** Magnified pictures of bipolar electrodes for attaching to the VN. **(C)** (From top to bottom) EMG signals, and their RMS; filtered (300–1,000 Hz) left VN signals, their spike rates (bin = 1 s); right VN signals, and their spike rates. Move periods were defined based on EMG RMS (green areas) and excluded from VN spike rate analyses (N/A). The timing indicated by the orange numbers is magnified in **D**. **(D)** Magnified EMG and VN traces, with red dots indicating detected VN spikes. **(E)** (Left) The peak-to-peak amplitude, defined as the difference between the positive and negative peaks, was computed for each VN spike. (Right) Distributions of spike amplitudes for the left and right VN (*n* = 9 rats). The black line represents the summarized data across all rats, and each gray line represents an individual rat. **(F)** Two representative traces of simultaneously recorded VN signals and ECG signals. Red dots indicate detected VN spikes. Vertical dotted lines represent R-peak timing from ECG signals.

We first examined the relationship between ECG-derived heart rate and VN spike rate (Supplementary Figures 1A,B; bin = 1 s). In all rats tested, spike rates in both the left and right VN were significantly and positively correlated with heart rate (Supplementary Figure 1C; *n* = 4 rats each, *p* < 0.05, Pearson correlation), indicating that bilateral VN activity increases occur during heart rate elevations that reflect enhanced sympathetic tone. Given that efferent parasympathetic activity would instead be associated with heart rate decreases, these results suggest that the recorded VN signals are not predominantly efferent parasympathetic in origin, but rather more likely represent afferent signals reflecting peripheral organ activity.

### The VN shows sparse and burst spikes

To further characterize spike timing dynamics at the millisecond timescale, we computed the distribution of inter-spike intervals (ISIs) (Figure 2A). For both the left and right VN, the ISI distribution appeared linear when plotted on logarithmic axes, consistent with a power-law distribution, indicating a scale-invariant property of spike timing. Furthermore, the coefficient of variation (CV) of inter-spike intervals over the entire recording period was markedly greater than 1 in both the left and right VN of all rats tested (Figure 2B; *n* = 9 rats each). No significant differences in CV were found between the left and right VN (*t*_8_ = 0.57, *p* = 0.58, paired *t*-test). These results indicate that VN spikes occur in an intermittent burst-like pattern. Given these intermittent burst-like spike profiles arising from the power-law distribution, we defined sparse spikes and burst events using arbitrarily chosen parameters to describe typical VN spike patterns. First, sparse single spikes with markedly long ISIs (~3 s) were occasionally observed (Figure 2C), occurring at a frequency of less than 0.4/min (Figure 2D), which accounted for <1% of total recorded spikes. Second, the majority of spikes occurred as dense burst events (Figures 2E,F), defined as more than 30 consecutive ISIs shorter than 100 ms. The average duration of burst events was 0.5 s and 0.8 s (Figure 2G), and their frequency was 0.42/min and 0.59/min for the left and right VN, respectively (Figure 2D).

**Figure 2:**
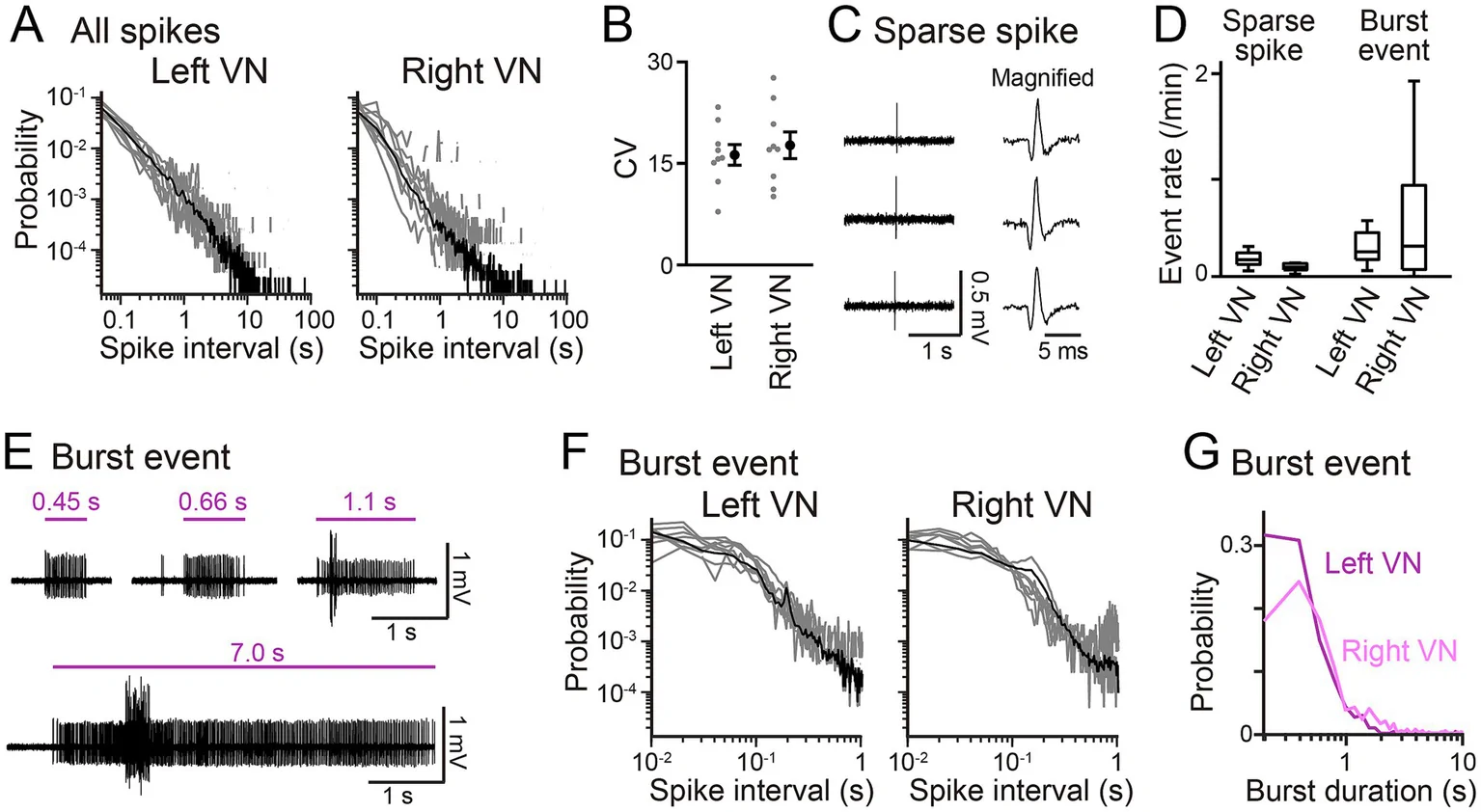
VN signals consist of sparse spikes and burst events. **(A)** Distributions of inter-spike intervals for the left and right VN (*n* = 9 rats). The black line represents the summarized data across all rats, and each gray line represents an individual rat. **(B)** The coefficient of variation (CV) of inter-spike intervals over the entire recording period, plotted for the left or right VN (mean ± SEM) (*n* = 9 each). Each gray dot represents each rat. **(C)** Representative sparse spike traces. **(D)** The frequency of sparse spikes and burst events in the left and right VN (*n* = 9 rats). **(E)** Representative burst event traces. The magenta bars above represent burst periods. **(F)** Distributions of inter-spike intervals within burst events for the left and right VN (*n* = 9 rats). The black line represents the summarized data across all rats, and each gray line represents an individual rat. **(G)** Distributions of burst event durations summarized from all rats (*n* = 9 rats).

### Left–right synchrony of VN spikes

Next, we examined whether and to what extent spike patterns of the left and right VN were synchronized (Figure 3A). First, the overall synchrony between the global temporal changes in spike rate of the left and right VN was quantified by cross-correlation analysis (Figure 3B; *n* = 9 rats). This correlation was computed by calculating the spike rates of the left and right VN in 1-s bins during non-movement periods and then computing the cross-correlation between them at 1-s lags. Overall, these correlation values varied significantly across time lags (*F*_8,64_ = 20.16, *p* = 8.1 × 10^−15^, one-way repeated measures ANOVA). The positive correlation value peaked at a time lag of 0 s, with an average of 0.30 ± 0.06, and correlation values at time lags from −1 to +1 s were significantly greater than zero (−1 s: *t*_8_ = 3.89, *p* = 0.016; 0 s: *t*_8_ = 4.97, *p* = 0.0049; +1 s: *t*_8_ = 4.18, *p* = 0.012, one-sample *t*-test with Holm correction). These results suggest that the left and right VN are synchronized on a timescale of seconds.

**Figure 3:**
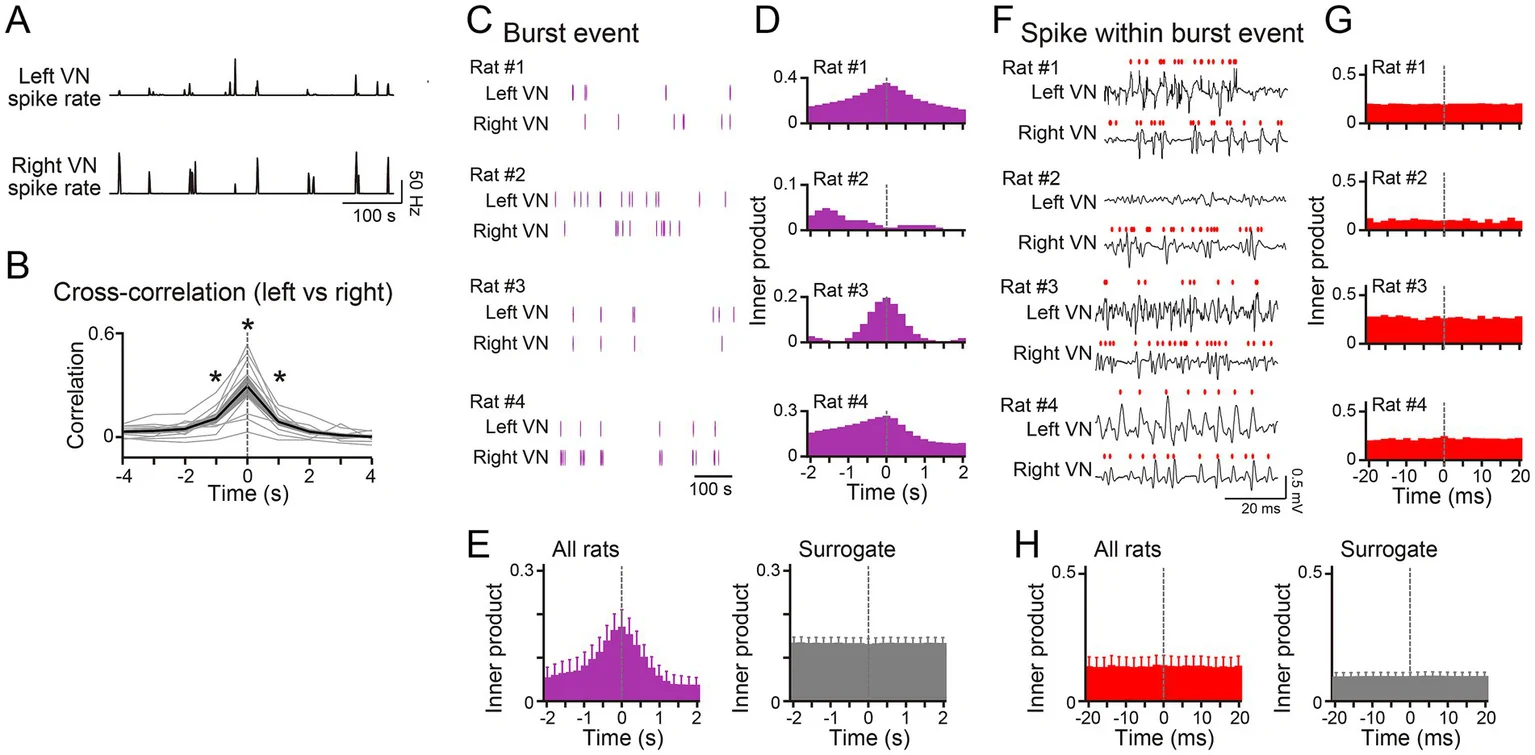
Left–right synchrony of VN spikes. **(A)** Typical spike rate changes in simultaneously recorded left and right VN in a rat. **(B)** Cross-correlograms of spike rate changes (bin = 1 s) between the left and right VN. The black line represents the average (*n* = 9 rats), and the gray areas represent SEM. Each gray line represents an individual rat. *p < 0.05, one-sample *t*-test with Holm correction versus zero. **(C)** Representative rastergrams of burst events (ticks) from the left and right VN in each rat. **(D)** Normalized inner products, computed from rastergrams of burst events in the left and right VN in the four rats corresponding to **C**, with the relative timing shifted across a range of time lags (bin = 0.2 s), analogous to cross-correlation. **(E)** (Left) Average normalized inner products across all rats (*n* = 9 rats). Error bars represent SEM. (Right) Normalized inner products computed from 1,000 surrogate datasets in which burst event timing was randomly shuffled. **(F)** Representative spike patterns within a burst event in the left and right VN. Red dots indicate detected spikes. **(G)** Normalized inner products, computed from rastergrams of spike patterns within burst events in the left and right VN in the four rats corresponding to **F**, with the relative timing shifted across a range of time lags (bin = 2 ms). **(H)** Average normalized inner products across all rats (*n* = 9 rats).

To further evaluate the left–right VN synchrony of burst events, we created raster plots in which the presence or absence of a burst event was represented as 1 or 0, respectively, for each rat (representative four rats shown in Figure 3C; bin = 0.2 s), and then computed the normalized inner product of these binary vectors across time lags from −2 to 2 s, analogous to cross-correlation analysis (Figure 3D). Overall, the average normalized inner product across all rats showed a modest peak at a time lag of 0 s (Figure 3E), which was significantly higher than those obtained from 1,000 surrogate datasets in which burst event timing was randomly shuffled (*p* < 0.05; *t*-test). This result demonstrates that burst events in the left and right VN were not always synchronized, but nonetheless exhibited a moderate degree of synchrony. Furthermore, we examined whether the spike patterns within these burst events were synchronized with millisecond-level precision. Representative spike patterns within a single burst event are shown in Figure 3F. Similar to the burst event analysis (Figure 3D), individual spike patterns within each burst event were converted into binary raster plots with 1 or 0 (bin = 2 ms), and the inner product of these binary vectors was computed across time lags from −20 to 20 ms (Figure 3G). Overall, the averaged inner product across all rats showed no apparent peak within this time window (Figure 3H). These results demonstrate that individual spike timing within burst events was not synchronized between the left and right VN with millisecond precision.

### VN spike patterns are not entrained to cycle-by-cycle gastrointestinal signals

Given that the majority of afferent VN fibers innervate the gastrointestinal tract, ongoing VN spike activity may be affected by the rhythmic dynamics of these peripheral organs. To test this possibility, we simultaneously recorded VN spike activity and gastric EGG and intestinal EEnG signals under urethane anesthesia conditions (Figures 4A,B). FFT analysis confirmed that the gastric EGG and intestinal EEnG signals exhibited peak power at frequencies of 0.07–0.11 Hz and 0.5–0.7 Hz, respectively (Figures 4C,E), consistent with their typical oscillatory motility cycles. To examine whether VN spikes were time-locked to the cycle-by-cycle gastrointestinal activity, we performed a phase analysis in which the cyclical EGG/EEnG signals were segmented into discrete phase bins, and the corresponding changes in VN spike rate were assessed across these phases (Figures 4D,F; bin = 1/10 π). To quantify phase-locking, Rayleigh’s *Z* values were computed for individual rats. In all rats, *Z* values were well below 3 (the threshold for significance at *p* < 0.05) (Figures 4D,F, right). These results suggest that ongoing VN spike activity is not strongly entrained to the oscillatory gastric/intestinal motility patterns on a cycle-by-cycle basis.

**Figure 4:**
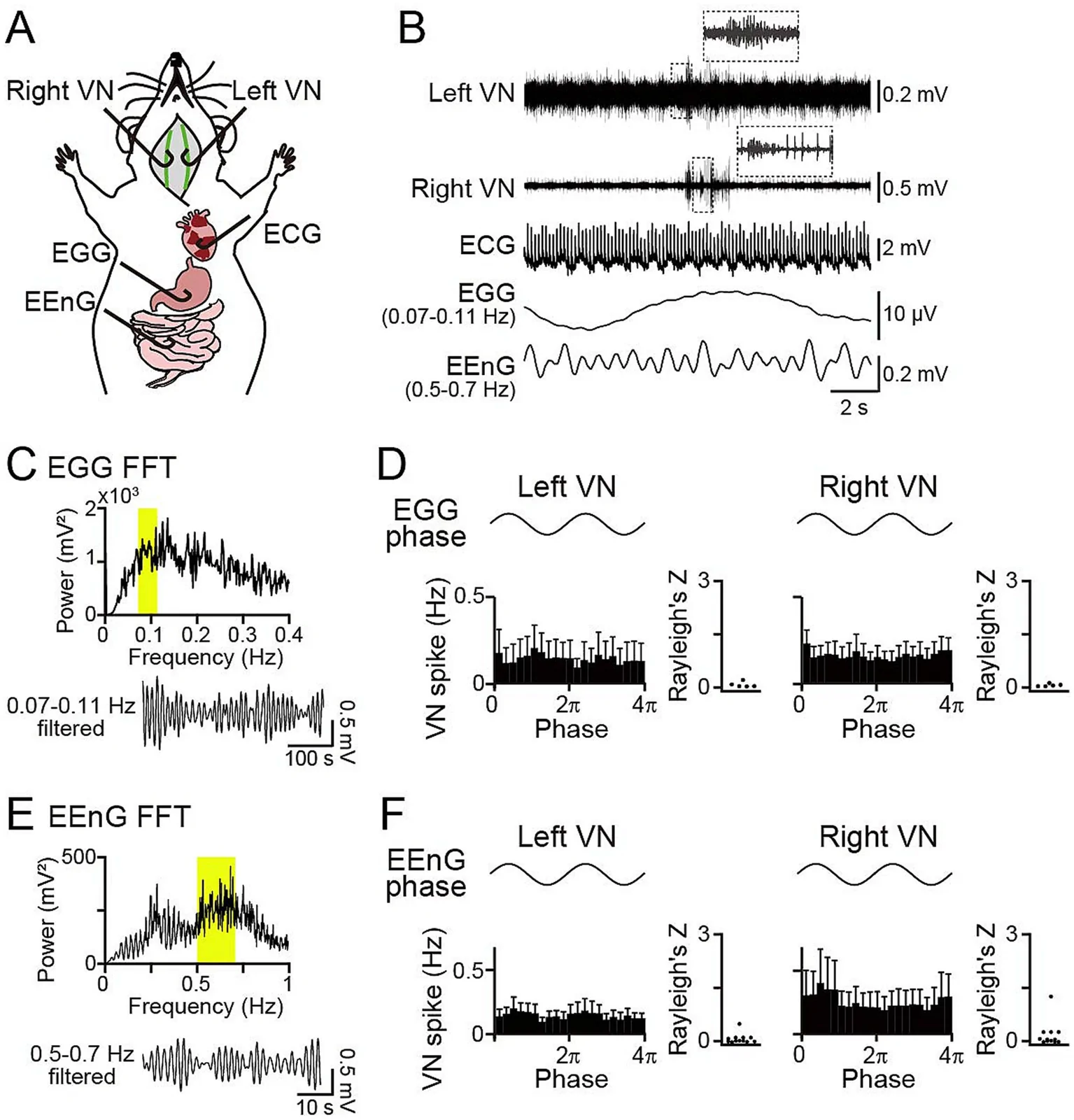
VN spikes are not time-locked to gastrointestinal activity on a cycle-by-cycle basis. **(A)** Simultaneous recordings of left and right VN signals and ECG, EGG, and EEnG in an anesthetized rat. **(B)** Representative traces of simultaneously recorded signals. **(C)** Fast Fourier transformation (FFT) of EGG signals (top) and representative filtered EGG signals at 0.07–0.11 Hz (bottom). The yellow area represents 0.07–0.11 Hz. **(D)** (Left) Relationship between EGG signal phase and VN spike rates (*n* = 9 rats). Error bars represent SEM. (Right) Rayleigh’s *Z* values computed from the datasets in the left panels. Each dot represents one rat. **(E)** FFT of EEnG signals (top) and representative filtered EEnG signals at 0.5–0.7 Hz (bottom). The yellow area represents 0.5–0.7 Hz. **(F)** Relationship between EEnG signal phase and VN spike rates (*n* = 5 rats).

### VN spikes are increased in response to enhanced peripheral organ function

While the results in Figure 4 showed no clear relationship between VN spikes and the oscillatory rhythms of individual peripheral organs, VN activity changes may be induced when peripheral organ function is dynamically altered. To directly assess this possibility, we applied receptor agonists to selectively evoke activity in each organ (Figure 5A). After recording baseline VN spike patterns for 30 min, acetylcholine (ACh) was topically applied to the intestine and stomach to enhance gastrointestinal motility, and isoproterenol (Iso) was topically applied to the heart to increase heart rate (Figure 5C). To prepare for the subsequent agonist application, the corresponding antagonist for the activated receptors was applied to the same organ to suppress the drug-evoked activity 15 min after the agonist application (Figure 5B; Atropine (Atr) for the intestine and stomach; Propranolol (Pro) for the heart). ACh application transiently increased gastrointestinal signal amplitude, peaking within ~1 min (Figure 5C, left and middle). Iso application gradually increased heart rate, reaching a peak at ~3 min (Figure 5C, right). Based on these temporal change patterns, VN spikes during the 1-min window following drug administration were analyzed by computing Z-scored spike rates based on baseline activity (1-min bins in the preceding 10 min). Z-scores exceeding 1.96 (*p* < 0.05) were considered significant. Of 15 rats tested, 11 showed significant increases in left VN spike rates in response to intestinal ACh application, 6 in response to stomach ACh application, and 9 in response to heart Iso application (Figure 5D; as indicated by red lines). Of 15 rats tested, 10 showed significant increases in right VN spike rates in response to intestinal ACh application, 7 in response to stomach ACh application, and 10 in response to heart Iso application (Figure 5E). These results demonstrate that pharmacologically enhanced gastrointestinal motility and increased heart rate are both capable of increasing bilateral VN spike rates. Comparisons of drug-induced spike rates between the left and right VN revealed that stomach ACh application evoked significantly higher spike rates in the left VN than in the right VN (*n* = 15 rats, *W* = 22, *p* = 0.030, Wilcoxon signed-rank test), whereas no significant left–right difference was observed in response to intestinal ACh application (*W* = 75, *p* = 0.42) or heart Iso application (*W* = 76, *p* = 0.15). These results suggest that gastric motility signals are preferentially conveyed through the left VN rather than the right VN.

**Figure 5:**
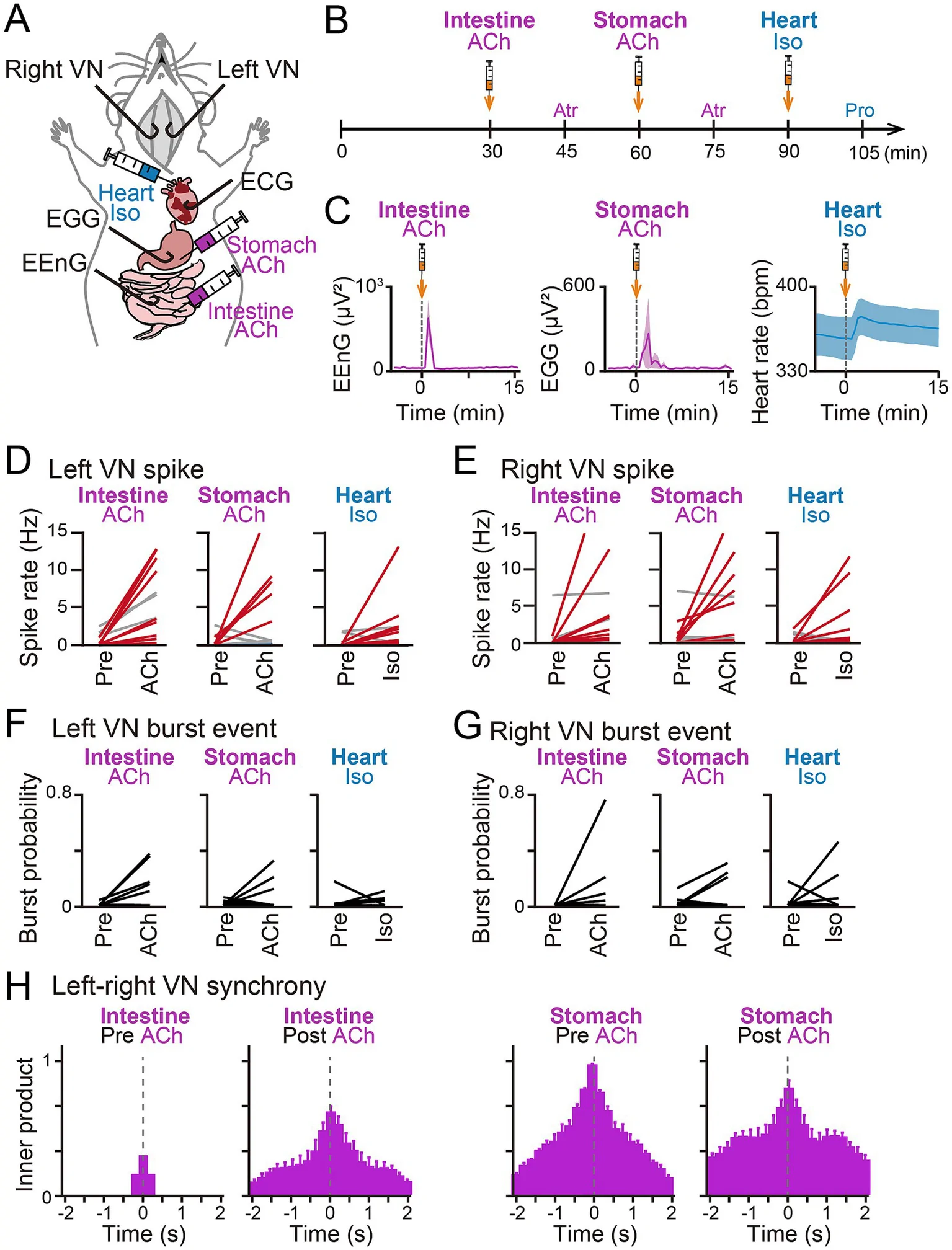
VN spikes are induced by increased gastrointestinal motility and heart rate. **(A)** During recording, drug was applied to each organ. **(B)** Time course of drug applications. **(C)** (From left to right) Changes in EEnG signal amplitude aligned to the onset of intestine ACh application (*n* = 6 rats), EGG signal amplitude aligned to the onset of stomach ACh application (*n* = 6 rats), and heart rates aligned to the onset of heart Iso application (*n* = 6 rats). Thick lines represent means and shaded areas represent SEM. **(D)** Changes in left VN spike rates in response to each drug application (*n* = 15 rats). Each line represents each rat and red lines indicate significant increases after drug applications (*p* < 0.01). **(E)** Same as **D** but for right VN spike rates. **(F,G)** Changes in burst event rates in response to each drug application in the left **F** and right **G** VN. (*n* = 15 rats). **(H)** Normalized inner products computed from burst events in the left and right VN, with the relative timing shifted across a range of time lags (bin = 0.2 s). Data were averaged across all rats (*n* = 15 rats).

Furthermore, we examined changes in burst events in the same datasets. With the same criteria as in Figure 2E, burst events were defined before and after drug application, and burst probability was computed as the proportion of time occupied by burst events relative to the total recording period. In a subset of recordings, burst events increased in both the left and right VN in response to each organ activation (Figures 5F,G). No significant left–right differences in burst events were observed in response to intestinal ACh application (*n* = 15 rats, *p* = 0.56), stomach ACh application (*p* = 1.00), or heart Iso application (*p* = 0.63, Wilcoxon signed-rank test). As a measure of left–right VN synchrony, the normalized inner product of binary vectors composed of left and right VN burst events was computed as in Figure 3E. They showed a modest peak at a time lag of 0 s following ACh application to the intestine and stomach (Figure 5H). Compared with the pre-application period, this peak value (−0.2 to +0.2 s) was significantly increased following intestinal ACh application (*t*_13_ = 2.47, *p* = 0.028, paired *t*-test), whereas no significant change was observed following stomach ACh application (*t*_12_ = 1.11, *p* = 0.29). These results suggest that left–right synchronized VN bursts are enhanced, at least when intestinal motility is increased. These analyses were not applied to the cardiac drug condition, as synchronous burst events between the left and right VN were too few to permit reliable analysis. These results demonstrate that enhanced gastrointestinal activity is sufficient to induce bilaterally synchronized burst events in the VN, a pattern resembling that observed under freely moving conditions.

### Changes in PFC LFP patterns during VN burst events

To examine how increased VN activity is related to brain activity states, we performed LFP recording from a cortical region in addition to the VN recording (Figure 6A). The PFC was selected as a representative cortical region because it is a higher-order area located multiple synapses away from peripheral inputs, and its activity may therefore reflect dynamics that generalize to other neocortical regions. In addition, the PFC is involved in a wide range of functions, including cognition, making it well suited for examining how VN activity may influence such higher brain functions. LFP signals in the 2–100 Hz band were converted into power spectra using wavelet analysis (Figure 6B) and LFP power was calculated separately for periods with and without VN burst events and was Z-scored relative to periods in which neither the left nor the right VN exhibited burst events. To visualize the relationship between VN burst events and LFP power changes, burst-event-triggered LFP spectrograms were constructed for left VN burst events, right VN burst events, and synchronized burst events (Figures 6C–E). When burst events emerged in the left VN, LFP power at frequencies of 2–100 Hz, except 10–20 Hz, was significantly increased compared with periods when neither the left nor right VN exhibited burst events (Figure 6C; *n* = 1,290 events from 9 rats; *p* < 0.05, paired *t*-test followed by FDR correction). When burst events emerged in the right VN, LFP power was significantly increased at 2–3 Hz and 20–100 Hz, whereas it was significantly decreased at 8–20 Hz (Figure 6D; *n* = 3,934 events from 9 rats; *p* < 0.05, paired *t*-test followed by FDR correction). When synchronous burst events emerged between the left and right VN, LFP power at 2–10 Hz and 40–100 Hz was significantly increased (Figure 6E; *n* = 517 events from 9 rats; *p* < 0.05, paired *t*-test followed by FDR correction). These results indicate that PFC LFP patterns are substantially modulated during VN burst events, potentially underlying vagal activity-mediated cognitive functions.

**Figure 6:**
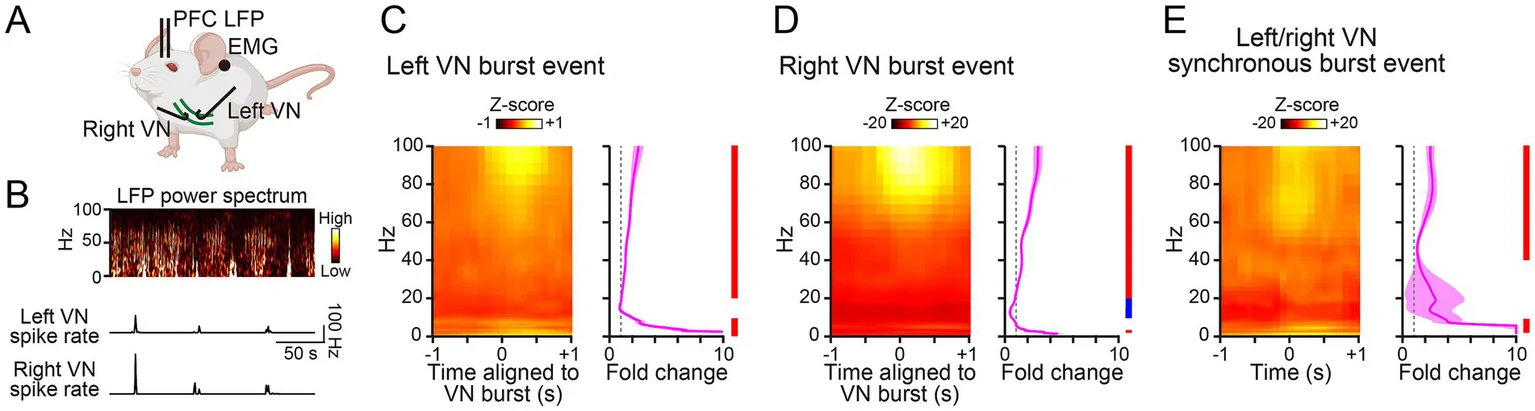
PFC LFP power is modulated during VN burst events. **(A)** Simultaneous bilateral VN and PFC LFP recordings. **(B)** Representative PFC LFP wavelet spectrum (2–100 Hz) and VN spike rate changes. **(C)** (Left) Average LFP power (*z*) spectrogram aligned to the onset of left VN burst events (*n* = 9 rats). (Right) Fold changes in LFP power during left VN burst events relative to that during periods without events, plotted against frequency bands (*n* = 1,290 events from 9 rats). The gray line represents 1. Shaded areas represent 95% confidence intervals. Red and blue bars on the right indicate frequencies with significant increases and decreases, respectively (*p* < 0.05, paired *t*-test with FDR correction). **(D)** Same as **C** but for right VN burst events (*n* = 3,934 events from 9 rats). **(E)** Same as in **C** but for left–right VN synchronous burst events (*n* = 517 events from 9 rats).

Brain oscillatory patterns correlate with slower activity of peripheral organs, such as gastric rhythms (Choe et al., 2021; Cao et al., 2022), and these peripheral–brain interactions are known to be mediated by the VN (Cao et al., 2022). To test whether a similar relationship between PFC LFP and EGG signals was present in our recordings, both signals were simultaneously recorded from anesthetized rats before and after bilateral vagotomy (Supplementary Figure 2A). PFC LFP-EGG coherence was computed at the dominant gastric frequency (0.07–0.11 Hz) and was significantly reduced following vagotomy (Supplementary Figure 2B; *n* = 5 rats; *t*_4_ = 4.80, *p* = 0.0086, paired *t*-test). These results demonstrate that coupling between prefrontal cortical activity and gastric rhythms is mediated by the VN.

Because cortical LFP signals provide a measure of sleep–wake states, we next examined how VN spike patterns varied across these states (Supplementary Figure 3A). Based on PFC LFP power in the delta (0.5–4 Hz), theta (6–10 Hz) and gamma (30–80 Hz) bands, individual 4-s epochs were classified into four behavioral states. EMG-defined non-movement periods (without green area in Supplementary Figure 3B) were classified as active wakefulness (AW), quiet wakefulness (QW), NREM sleep, and REM sleep (Supplementary Figure 3B; for more detail, see Materials and Methods) (Scheffzuk et al., 2011; Watson et al., 2016). The majority of non-movement periods were classified as quiet wakefulness and NREM sleep periods (Supplementary Figure 3C; *n* = 9 rats). Overall, spike rates in both the left and right VN were significantly higher during active wakefulness than during the other states (Supplementary Figure 3D; active wakefulness vs. other periods, *p* < 10^−10^, Mann–Whitney U test followed by Bonferroni correction). These results suggest that the recorded VN activity is greater during active wakefulness, even in the absence of overt movement, than during NREM or REM sleep states.

## Discussion

In this study, we performed simultaneous electrophysiological recordings of left and right VN spike patterns in rats. We found that ongoing spike patterns in both the left and right VN during non-movement periods were predominantly characterized as burst events. Some, but not all, VN burst events were temporally synchronized, whereas spike timing within individual bursts showed no apparent synchronization. PFC LFP power across the 2–100 Hz frequency range was substantially modulated during VN burst events. Under anesthetized conditions, VN spike timing was not prominently entrained to oscillatory gastrointestinal motility on a cycle-by-cycle basis. Pharmacological enhancement of gastrointestinal activity was sufficient to induce bilaterally synchronized VN burst events. A limitation of this study is that the recordings were obtained from the cervical VN and therefore contain both afferent and efferent components. Given that 75%–90% of cervical VN fibers are afferent (Evans and Murray, 1954; Agostoni et al., 1957; Prechtl and Powley, 1990; Miki et al., 2025), and assuming no substantial difference in firing rates between the two fiber types, the majority of the recorded signals is likely to reflect afferent activity. This view is further supported by the pharmacological experiments targeting individual organs, suggesting that the recorded signals are largely afferent in origin.

Our recording confirmed that burst spikes dominated both left and right VN overall activity, which are thought to reflect physiological states of various peripheral organs including the digestive system, cardiovascular system, and respiratory system (Quigg, 1991; Berthoud et al., 1997; Wang and Powley, 2000; Kosta et al., 2010; Fontaine et al., 2021; Lovelace et al., 2023; Wang and Tache, 2024; Lansbury et al., 2025; Liu et al., 2026). Indeed, increases in burst spike patterns were replicated by pharmacological activation of the gastrointestinal tract. An important observation is that, regardless of whether they occurred spontaneously or were artificially evoked from organs, such burst spikes did not persist continuously (e.g., 1-min periods in which peripheral organ activation was induced by pharmacological manipulation), but occurred intermittently with intervening silent periods. Several mechanisms may underlie such intermittent spike patterns: intrinsic spike-generating properties of the VN, including the time constants of ion channel gating and refractory periods, or dynamic changes in the chemical or physical signals detected by VN terminals at the peripheral organs. In addition, our evidence that VN activity is mainly composed of intermittent bursts provides important implications for the design of vagus nerve stimulation (VNS) parameters, which are widely used in both clinical studies in humans and basic research in animals. Because the temporal parameters of VNS offer a high degree of freedom, a wide range of protocols has been proposed, from tonic stimulation to temporally patterned stimulation consisting of intermittent short bursts as reviewed in previous reports (Thompson et al., 2021; Austelle et al., 2024). The present results suggest that temporally patterned stimulation may be more effective, as it more closely recapitulates the intrinsic and endogenous VN activity.

VN burst spike timing was found to be partially synchronized between the left and right VN. These physiological activities are considered to arise from anatomical patterns in which the projection patterns of the left and right VN onto peripheral organs, such as the heart and gastrointestinal tract (Quigg, 1991; Kosta et al., 2010; Fontaine et al., 2021; Neuhuber and Berthoud, 2021; Bassi et al., 2022; Lovelace et al., 2023; Liu et al., 2026), partially overlap, while some local regions receive exclusive vagal innervation from only one side (Neuhuber and Berthoud, 2021; Bassi et al., 2022). Furthermore, the left and right VN differ in their receptor types and in their mechanisms for sensing chemical or physical signals (Zhao et al., 2022; Lansbury et al., 2025; Ritchey et al., 2026). These facts imply that the partially overlapping anatomical projection patterns of the left and right VN contribute to synchronized burst spike patterns, while side-specific mechanisms may selectively activate only one side. Overall, such heterogeneous spike patterns are considered to increase the capacity of the VN to detect a broader range of organ activity states, constituting an advantageous interoceptive system.

Temporally concentrated VN burst spikes, compared with temporally sparse spikes, are considered an efficient activity pattern for enhancing temporal summation of synaptic input onto nucleus tractus solitarius neurons and further downstream brain neurons. Specifically, one-side burst events confined to the right or left VN, or synchronized bilateral events could produce spatially distinct effects, such as additive integrating effects or more selective responses by inhibiting the other side, thereby producing a diverse repertoire of information processing modes in the brain.

Our PFC LFP analyses showed that burst spiking in either unilateral or bilateral VN was associated with increased or decreased LFP power across several frequency bands. Delta (~2 Hz) and gamma (>30 Hz) power in the PFC LFP commonly increased when either left or right VN burst events occurred. Assuming that the recorded VN activity is predominantly afferent, afferent VN burst events may act as salient inputs that shape PFC power dynamics. These inputs are considered to be reflected in an increase in delta power, which may in turn drive local excitation–inhibition shifts, as reflected in the subsequent increase in gamma power. In contrast, theta and alpha (~10–20 Hz) power decreased selectively during right VN burst events. These power change patterns in the PFC suggest that right VN burst events promote a transition in the cortex toward a more flexible processing mode for salience-driven local computation. Conversely, assuming that the recorded VN activity likely includes a minor efferent component, the observed PFC LFP power states may also contribute to the induction of a parasympathetic-dominant physiological state in peripheral organs. Determining whether these interactions are mediated by afferent or efferent signaling will require recordings that selectively resolve each VN component.

Building upon the complex projection patterns of the VN revealed by anatomical studies, the present study demonstrated that the left and right VN exhibit physiological activity characterized by both left–right spike synchronization and independent unsynchronized spike patterns. Such diverse VN spike activity patterns are likely to influence a wide range of VN-mediated functions, including not only reflexive homeostatic regulation but also higher brain functions such as emotion, memory, and adaptation to varying contextual demands. Future studies focusing on the coordination of the left and right VN are expected to further advance our understanding of the diversity and precision of brain–body interactions.

## Data Availability

The original contributions presented in the study are included in the article/Supplementary material, further inquiries can be directed to the corresponding author.
