# Supplementary material for: Left–right synchronicity of vagal spikes and their coordination with peripheral and cortical activity in rats
Source: Front Neural Circuits. 2026 Oct 30;20:1944995. doi: 10.3389/fncir.2026.1944995 (PMC13759806; doi:10.3389/fncir.2026.1944995)
Supplement: Supplementary file 1 [file Data_Sheet_1.pdf]

Supplementary figures

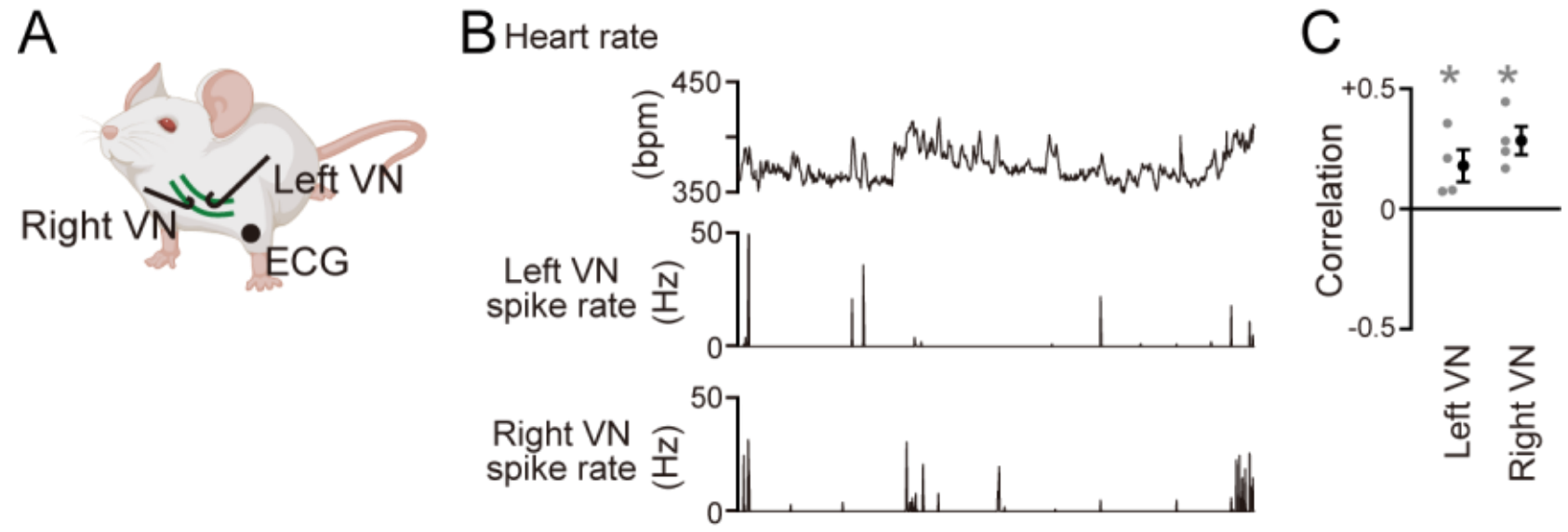

Supplementary Figure 1 Correlation between heart rates and VN spike rates. (A) Simultaneous recordings from left and right VN signals and ECG signals in a freely moving rat. (B) (From top to bottom) Heart rates, left VN spike rates, and right VN spike rates (bin = 1 s) during a non-movement period. (C) Correlation between heart rates and VN spike rates, plotted for the left or right VN (mean ± SEM) (n = 4 each). Each gray dot represents each rat.

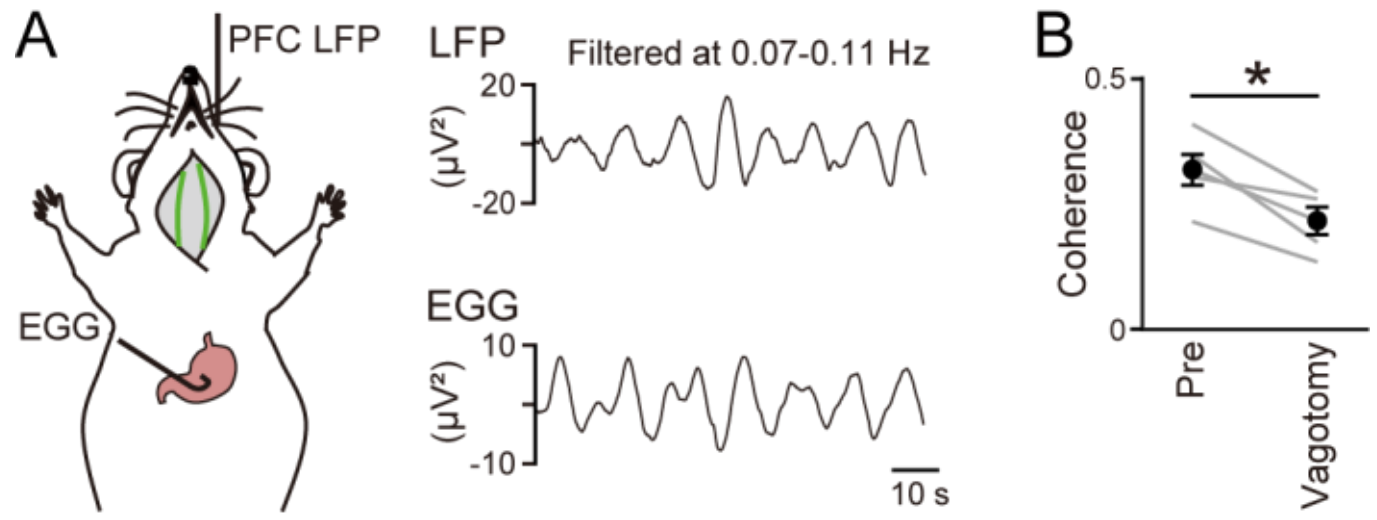

Supplementary Figure 2 Vagotomy reduces coherence between PFC LFP and EGG signals. (A) Simultaneous recording of PFC LFP and EGG signals filtered at 0.07–0.11 Hz in an anesthetized rat. (B) Changes in coherence between PFC LFP and EGG signals before and after vagotomy (mean ± SEM) (*n* = 5 rats, **P* = 0.0086, paired *t*-test). Each gray line represents each rat.

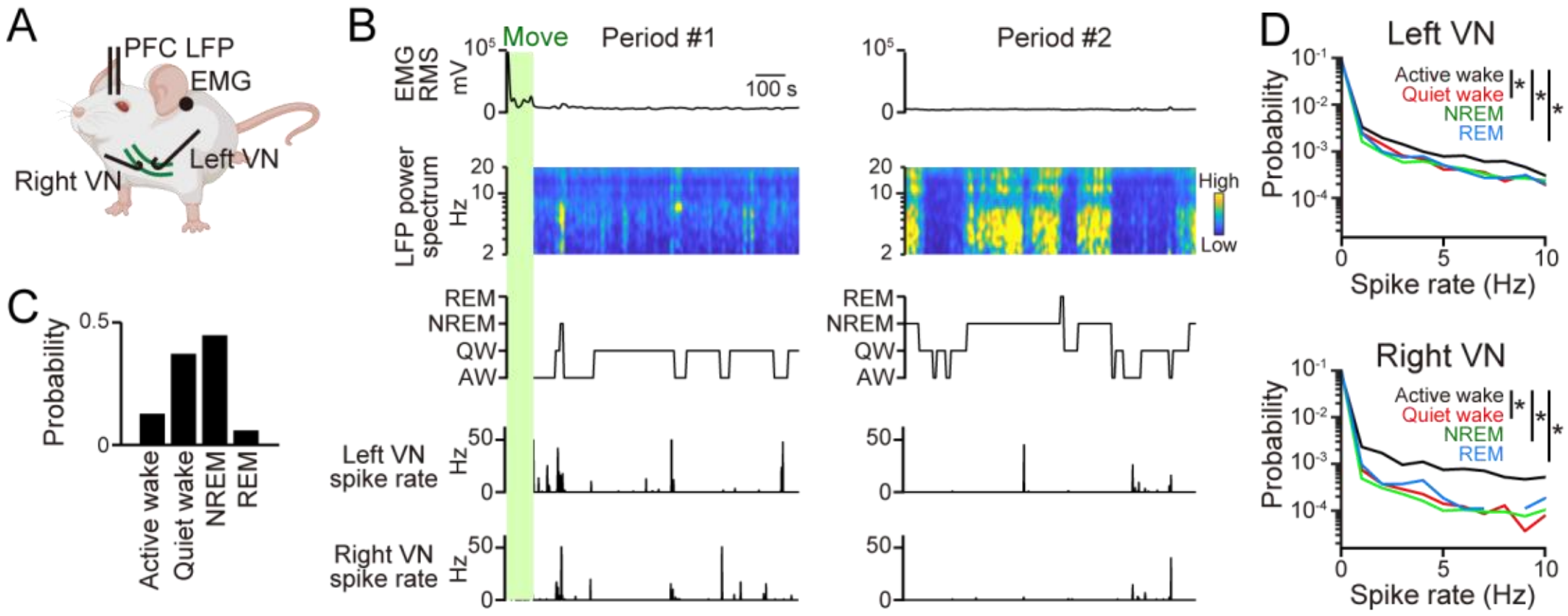

Supplementary Figure 3 Vagus nerve activity and cortical LFP-based sleep state classification. (A) Simultaneous bilateral VN and PFC LFP recordings. (B) Two representative periods. (From top to bottom) EMG RMS, PFC LFP wavelet spectrum (2–20 Hz), LFP-based sleep states, left VN spike rates, and right VN spike rates (bin = 1 s). Move periods were defined based on EMG RMS (green areas) and excluded from analyses. (C) Proportion of time in each behavioral state (*n* = 9 rats). (D) Distributions of spike rates (bin = 1 s) for the left and right VN in each sleep state, summarized data across all rats (*n* = 9 rats). *$P < 10^{-10}$, Mann–Whitney U test followed by Bonferroni correction.
